# Safety and Efficacy of the Ayurvedic Formulation Guduchi Ghana Vati as a Preventive Remedy in COVID-19

**DOI:** 10.7759/cureus.58807

**Published:** 2024-04-23

**Authors:** Sanjeev Sharma, Pawankumar Godatwar, Manohar Pareek, HML Meena, Anand Sharma, Sanjay Tamoli

**Affiliations:** 1 Ayurvedic Surgery, National Institute of Ayurveda, Jaipur, IND; 2 Ayurvedic Pathology, National Institute of Ayurveda, Jaipur, IND; 3 Ayurveda and Indian System of Medicine (ISM), Government of Rajasthan, Jaipur, IND; 4 Ayurvedic Medicine, National Institute of Ayurveda, Jaipur, IND; 5 Ayurvedic Pathology, Directorate of Ayurveda, Ajmer, IND; 6 Research, Target Institute of Medical Education and Research, Mumbai, IND

**Keywords:** guduchi, ayurveda, severity of infection, number of episodes, healthy volunteers, covid-19 prevention, tinospora cordifolia

## Abstract

Background and objectives

Guduchi (*Tinospora cordifolia*) is a well-known Ayurvedic herb used as a preventive and curative remedy for various infections and immunity-related conditions. This study aimed to evaluate Guduchi Ghana Vati as a preventive remedy for COVID-19 and non-COVID-19 infections in a healthy population.

Materials and methods

An open-labeled, multi-centric, randomized, comparative, interventional, prospective community-based clinical study was conducted on healthy individuals at the community level in five different districts of Rajasthan by the National Institute of Ayurveda (NIA), Jaipur, India. Participants were divided into two groups. One group received Guduchi Ghana Vati as an intervention for 45 days, and the control group did not receive any intervention. Incidences of COVID-19 infection, non-COVID-19 infections, their severity, and hospitalization requirements were assessed. Safety was evaluated through monitoring of adverse reactions.

Results

Among the 10,022 participants who completed the study, the incidence of COVID-19 infection was found to be lower in those taking Guduchi Ghana Vati compared to the control group; however, the difference was statistically non-significant. The severity of COVID-19 based on the WHO ordinal scale was found to be significantly lower in the Guduchi Ghana Vati group compared to the control group. The number of episodes and severity of non-COVID-19 illness were also significantly lower in participants taking Guduchi Ghana Vati compared to the control group. No major adverse drug reactions were observed.

Conclusion

Guduchi Ghana Vati has the potential to act as a safe and effective remedy for the prevention of infection and immunity-related conditions, including COVID-19.

## Introduction

The sudden outbreak of COVID-19, pneumonia of unknown origin, first started in Wuhan, in the Hubei Province of China, in December 2019. The causative virus, severe acute respiratory syndrome coronavirus-2 (SARS-CoV-2), rapidly spreads amongst the human population through close contact by inhaling contaminated air and small droplets containing the virus released by cough, sneezing, or breathing [1, 2]. The World Health Organization named it coronavirus disease 2019 (COVID-19) and declared the disease a pandemic [3]. The majority of patients with COVID-19 are asymptomatic and recover without any major interventions. Few patients have fever, muscle pains, headaches, sore throats, loss of taste, anosmia, diarrhea, sneezing, colds, coughs, and breathing difficulties. Very few patients suffer from severe disease and may require hospitalization and critical care, including antiviral agents, glucocorticoids, monoclonal antibodies, antimalarial drugs, oxygen, ventilators, and other life-supportive therapy. A small number of patients may die due to severe cardiorespiratory or other complications [4].

Regular hand washing, cleaning with soap and water or sanitizer, especially after touching a surface in public places, using masks and gloves, covering the mouth and nose while coughing and sneezing, not touching the face with unclean hands, and social distancing are the preventive measures advised to contain the spread of COVID-19 [5]. As preventive measures, the consumption of Ayurvedic medicines such as Chyavanprasha, Agastya Haritaki, and Brahma Rasayana, along with the use of haldi (turmeric), has been widely suggested. Also, the consumption of herbs like Ashwagandha (*Withania somnifera*), Guduchi (*Tinospora cordifolia*), Tulsi (*Ocimum sanctum*), Maricha (*Piper nigrum*), Shunthi *(Zingiber officinale*), and Pippali (*Piper longum*) has been advised as preventive measures [6,7]. *Tinospora cordifolia* (Guduchi) has been used as a rejuvenating drug (Rasayana) and tonic (Balya) in the management of age-related disorders [8]. Various studies have found that *Tinospora cordifolia* acts as an immunomodulator [9,10]. *Tinospora cordifolia* possesses free radical-scavenging capacity. It decreases oxidative stress by increasing glutathione and other anti-oxidant enzymes and down-regulating proinflammatory cytokines [11]. *Tinospora cordifolia* stimulates helper T-cell immune and innate immune responses and develops antigen-specific immunity [10]. It also increases acetylcholine, which is responsible for enhancing cognitive function [10]. *Tinospora cordifolia* also possesses antimicrobial, anti-inflammatory, anti-stress, and anxiolytic activities [9,10]. Based on these properties of Guduchi, it may be said that Guduchi may be useful in improving immunity and thus reducing the incidence of infections and allergies. These may lead to improved health status in healthy individuals, helping them to fight against viral infections.

Guduchi has also been listed in the official advisory of the Ministry of AYUSH (short for Ayurveda, Yoga, Naturopathy, Unani, Siddha, and Homeopathy) of the Government of India as an immunity-boosting herb for the possible prevention of COVID-19. Therefore, the present clinical study was planned to evaluate the efficacy and safety of Guduchi Ghana Vati as a preventive remedy for healthy individuals during the COVID-19 pandemic.

This article was previously posted to the Research Square preprint server on April 27, 2022.

## Materials and methods

Ethics

The study conformed to the principles of the Declaration of Helsinki and was approved by the institutional ethics committee (IEC), National Institute of Ayurveda (NIA), Jaipur, Rajasthan, India, on May 22, 2020. The study was registered with the Clinical Trials Registry of India (CTRI) on May 31, 2020, with the registration number CTRI/2020/05/025488. Participation in the study was voluntary, and confidentiality was ensured. Written informed consent was obtained from all study participants.

Study design

The study was conducted as an open-labeled, multi-centric, randomized, comparative, interventional, prospective community-based clinical study on healthy individuals. The primary objective of the study was to assess the incidence of COVID-19 in participants taking Guduchi Ghana Vati (Guduchi group) and those not taking it (control group) over 45 days. Secondary objectives included a comparative assessment of the incidence of other non-COVID-19 infections between the participants in the two groups. Assessment was also done concerning the severity and requirement of hospitalization, number of days in hospital, ICU admission, oxygen requirement, ventilator support, and mortality in those diagnosed with COVID-19. The severity of COVID-19 was graded as per the ordinal scale for clinical improvement of COVID-19 published by the WHO (Table 1) [12]. A global assessment of overall change as per the investigator and a safety assessment by evaluating the occurrence of adverse reactions were also done.

**Table 1:** The WHO ordinal scale for clinical improvement of COVID-19 disease Source: [12] RRT: renal replacement therapy; ECMO: extracorporeal membrane oxygenation

Patient state	Descriptor	Score
Uninfected	No clinical or virological evidence of infection	0
Ambulatory	No limitation of activities	1
	Limitation of activity	2
Hospitalized with mild disease	Hospitalized, no oxygen therapy	3
Hospitalized with severe disease	Oxygen by mask or nasal prongs	4
	Non-invasive ventilation or high-flow oxygen	5
	Intubation and mechanical ventilation	6
	Ventilation + additional organ support (pressers, RRT, ECMO)	7
Dead	Death	8

Sample size

A total of 10,022 participants were enrolled in this study. Those were divided into two arms, with 5,009 participants in the Guduchi group and 5,013 in the control group. No specific sample size calculation method was used to arrive at the desired number, considering the pandemic and carrying out the study on a community basis on a novel disease. However, subjects were equally distributed using the stratified block randomization technique.

Inclusion criteria

Healthy male and female participants between the ages of 18 and 70 (both inclusive), not having any acute or chronic medical/surgical condition that required either immediate or continuous medical monitoring or treatment, and who were ready to provide written informed consent and to willingly participate in and follow the protocol requirements of the study, were recruited.

Exclusion criteria

Participants already diagnosed with the COVID-19 infection, either recently or in the past, were excluded from the study. Participants with immune-compromised status, those on steroid treatment or any immunosuppressive therapy, were not considered for recruitment in the study. Participants participating in any other clinical studies or having participated in any other studies three months before screening in the present study and those with any other condition that, in the opinion of the investigator, made the patient unsuitable for enrolment were excluded from the study. Participants taking any Ayurvedic medicines or immunity-enhancing substances like chyawanprash, kadha, etc. were excluded from the study. Pregnant and lactating women were also excluded from the study.

Investigational product

The study intervention was Guduchi Ghana Vati, 500 mg tablets. Guduchi Ghana Vati was prepared as tablets from an aqueous extract of the stem of Guduchi (*Tinospora cordifolia*). The same was standardized to not less than 2.5% bitters with an herb extract ratio of 10:1. The Guduchi group was asked to take two tablets of Guduchi Ghana Vati twice a day with water for 45 days. The control group did not receive any intervention.

Study conduction

The study was carried out at the community level in five different districts, viz., Jaipur, Ajmer, Kota, Tonk, and Jodhpur, in Rajasthan, India. Healthy male and female participants were screened for eligibility criteria. Before recruiting participants in the study, informed consent was obtained (either through an e-consent process or written consent). Most participants provided e-consent after reading the complete details of the study through a specially prepared e-consent app, which included a participant information sheet (PIS) and informed consent form (ICF), which was 21 Code of Federal Regulations (CFR) compliant and as per Good Clinical Practice (GCP) guidelines. The e-consent app was used considering the pandemic guidelines of lockdown, social distancing, and the large population involved in the study. All participants were evaluated and recruited based on the inclusion and exclusion criteria of the study. No laboratory tests were performed during the screening visit, and all clinical findings were based on clinical histories provided by the participants and assessments by the investigator. The study participants were divided into the Guduchi group and the control group based on a computer-generated randomization list. Participants in the Guduchi group were advised to take Guduchi Ghana Vati at a dose of two tablets twice a day for 45 days, while participants in the control group were not given any study intervention but were followed up like the intervention group. Both groups were advised to follow their normal routine related to diet and activities, which they were following.

All the participants were asked to follow COVID-19-related guidelines like social distancing, wearing of masks, sanitization of hands, etc. (for prevention and containment of infection) as guided by the local health authorities and other government agencies from time to time. Participants were also asked to undergo tests for COVID-19 (or other conditions) as per the prevailing guidelines of local health authorities and the instructions of their treating physician and the investigator. Participants were provided with a mobile app-based e-diary to record their episodes of illness (COVID-19, non-COVID-19 infections, etc.) daily. The severity of the condition, the requirement of hospitalization, tests done (for COVID-19 or others), and treatment taken were also recorded through the e-diary by the participant. The e-diary was linked to an electronic case report form (e-CRF) to record and document the above information during the entire study period and was available to the research team and investigator. The e-diary was also used by the participants to record their daily consumption of study medication in the Guduchi group. A small number of participants who were unable to use e-diaries were provided with paper diaries to get the required information.

Telephonic communication was used for continuous follow-up with the participants during the entire study. At the end of the study (after 45 days), participants were asked to visit the study site, or the study personnel visited the subject to record his/her health condition and study-related parameters. A digital telecommunication system was created for recording regular follow-ups from the participants from baseline until the end of the study. A record of the telephonic communication was done at every 15-day interval from the baseline visit until 45 days in the e-CRF. The incidence of COVID-19 or non-COVID-19 infections, their seriousness, hospitalization, and treatment were noted, and the occurrence of any adverse event was also recorded in the e-CRF.

Statistical analysis

The study data that were generated and collected were put into statistical analysis to reach the results and conclusions. Demographic data are presented as mean±SD and percentage. The data obtained in the studies were subjected to appropriate statistical tests to test their significance. GraphPad InStat version 3.6 software (GraphPad Software, La Jolla, CA) was used for statistical analysis of the data. A p-value of <0.05 was considered significant.

## Results

Participants flow

**Figure 1:**
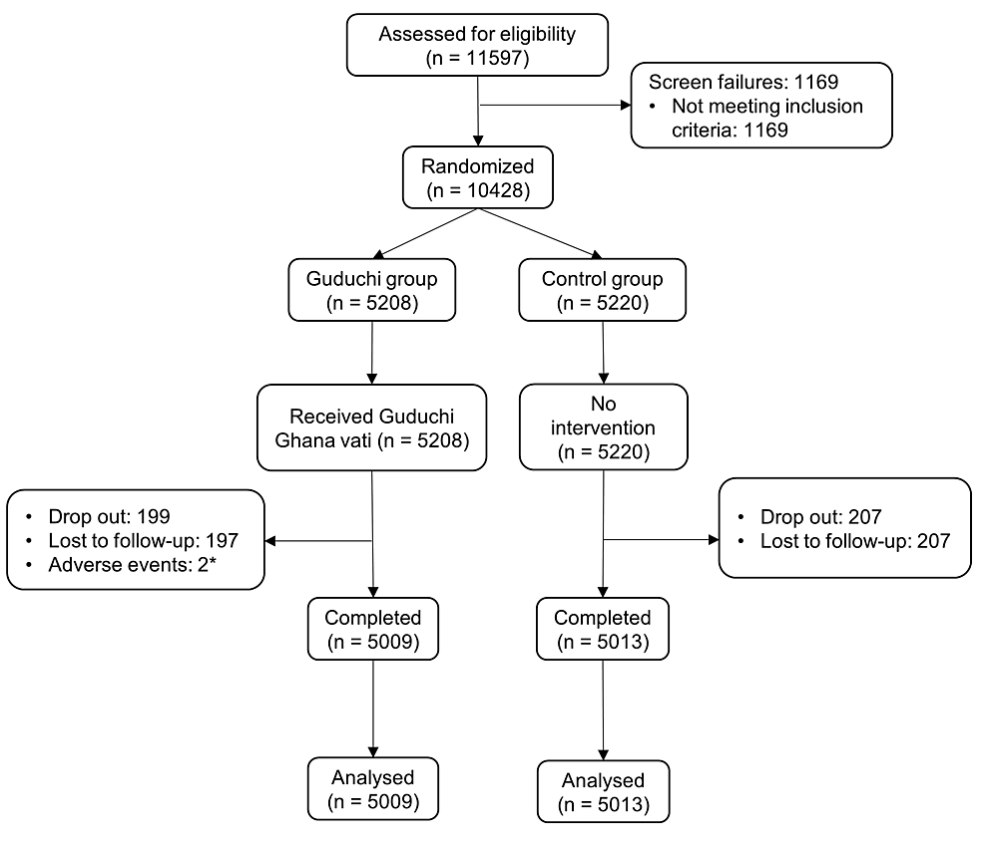
Flow of participants in the study *Two participants in the Guduchi group dropped out due to the occurrence of adverse events, which were possibly related to the consumption of the study product. These two events were of a mild nature, did not require any treatment, and resolved on their own.

A total of 11,597 participants were screened in the study, of whom 1,169 were screen failures, and 10,428 participants (5,208 in the Guduchi group and 5,220 in the control group) were recruited. There were 406 dropouts (199 in the Guduchi group and 207 in the control group) in the study. The reason for dropping out was primarily the loss of follow-up. Two participants in the Guduchi group dropped out due to the occurrence of adverse events, which were possibly related to the consumption of the study product. These two events were mild, required no treatment, and resolved on their own. A total of 10,022 participants completed the study (5,009 in the Guduchi group and 5,013 in the control group). The flow of participants in the study is presented in Figure 1.

Demographic details

There were 495 (9.88%) and 475 (9.48%) healthcare workers in the Guduchi group and the control group, respectively. In the Guduchi group, 32 (0.64%) were security/police staff, whereas in the control group, 15 (0.30%) were security/police staff. There were 719 (14.35%) and 695 (13.86%) housewives in the Guduchi group and the control group, respectively. There were 926 (18.49%) and 1,238 (24.70%) students in the Guduchi group and the control group, respectively. In the Guduchi group, 12 (0.24%) participants were sanitation/cleaning workers, whereas six (0.12%) participants were sanitation/cleaning workers in the control group; 111 (2.22%) participants in the Guduchi group were retired personnel, while 82 (1.64%) retired personnel were present in the control group. In the Guduchi group, 2,714 (54.18%) participants were from other professions/occupations like service, business, etc., whereas this number was 2,502 (49.91%) in the control group. The sex-wise distribution showed that there were 3,294 (65.76%) males and 1,715 (34.24%) females in the Guduchi group, while there were 2,928 (58.41%) males and 2,085 (41.59%) females in the control group. The mean age of participants in the Guduchi group was 38.33±12.06 years, while for those in the control group, it was 35.70±11.60 years, showing no significant difference between the groups. The baseline characteristics of the participants are summarized in Table 2.

**Table 2:** Baseline characteristics of the participants ^a^Each value is represented as numbers (percentages); ^b^Each value is represented as mean±SD of n=5,208 in the Guduchi group and 5,220 in the control group.

Demography	Guduchi group	Control group	p-value between the groups
Males, n (%)^a^	3294 (65.76)	2928 (58.41)	p>0.05
Females, n (%)^a^	1715 (34.24)	2085 (41.59)	p>0.05
Age (years)^b^	38.33±12.06	35.70±11.60	p>0.05

Effect on the incidence of COVID-19 and non-COVID-19-related illness

A total of 341 participants (3.40%) from both groups reported episodes related to infection like fever, sore throat, cough, cold, body aches, etc., of which 151 participants (1.50%) underwent testing for COVID-19 by real-time reverse transcription-polymerase chain reaction (RT-PCR). In the Guduchi group, of the 159 (3.17%) participants who had infection-related episodes, 77 (1.53%) underwent an RT-PCR test, of which 17 (0.34%) participants tested positive and 60 (1.19%) participants tested negative for COVID-19. In the control group, a total of 182 (3.63%) participants had infection-related symptoms, of which 74 (1.47%) participants underwent an RT-PCR test, of which 26 (0.52%) participants tested positive, and 48 (0.95%) participants tested negative for COVID-19. While a lower incidence of COVID-19 was observed in the Guduchi group, the same was found to be statistically non-significant (p>0.05).

**Figure 2:**
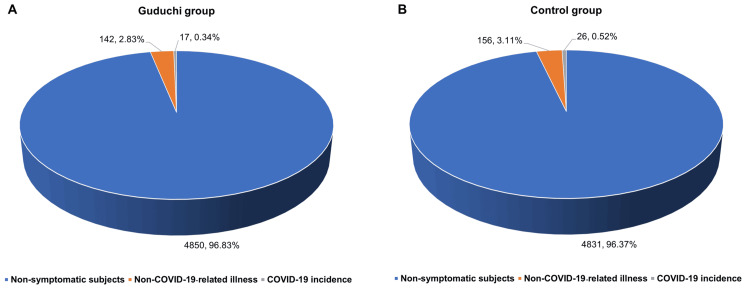
Incidence of COVID-19 and non-COVID-19-related illnesses Values are presented as numbers and percentages. No significant (p>0.05) difference was observed in the incidence of COVID-19 and non-COVID-19-related illnesses between the Guduchi group (Figure 2A) and control group (Figure 2B).

Of the 10,022 participants, a total of 298 (2.97%) were reported to have non-COVID-19-related illnesses in both groups, with 142 (2.83%) in the Guduchi group and 156 (3.11%) in the control group. Though a lesser number of participants reported non-COVID-19-related illnesses, the difference between groups was statistically non-significant (p>0.05). The results of the incidence of COVID-19 and non-COVID-19-related illnesses in the Guduchi group and control group are shown in Figures 2A-2B. 

Effect on the severity of COVID-19

**Figure 3:**
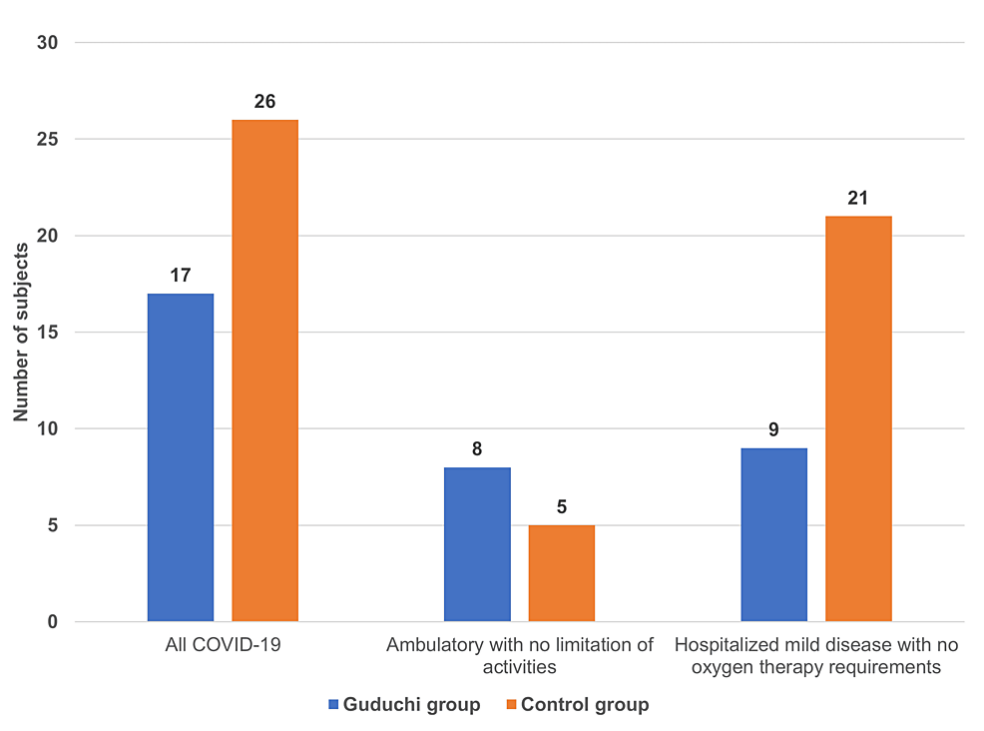
The severity of COVID-19 infection among the groups The severity of COVID-19 infection was significantly (p<0.05) lesser in the Guduchi group compared to the control group. The WHO ordinal scale was used to assess the severity of the COVID-19 infection.

Out of the 17 participants who tested positive for COVID-19 in the Guduchi group, eight participants were categorized as “ambulatory" status (no limitation of activities) (score one each), while the remaining nine participants were categorized as “hospitalized with mild disease" status (no oxygen therapy) (score three each) as per the ordinal scale for clinical improvement of COVID-19 published by the WHO [12]. Out of the 26 participants who tested positive for COVID-19 in the control group, five were categorized as “ambulatory" status (no limitation of activities) (score one each), while the remaining 21 participants were categorized as “hospitalized with mild disease" status (no oxygen therapy) (score three each). The results signify a higher WHO ordinal at the time of COVID-19 diagnosis in the control group (score of 68) compared to the Guduchi group (score of 35), and the severity of COVID-19 infection in the Guduchi group was significantly (p<0.05) lesser compared to that of the control group. Details on the number of subjects with COVID-19 of different severity are shown in Figure 3.

Effect on the severity of non-COVID-19-related illness

**Figure 4:**
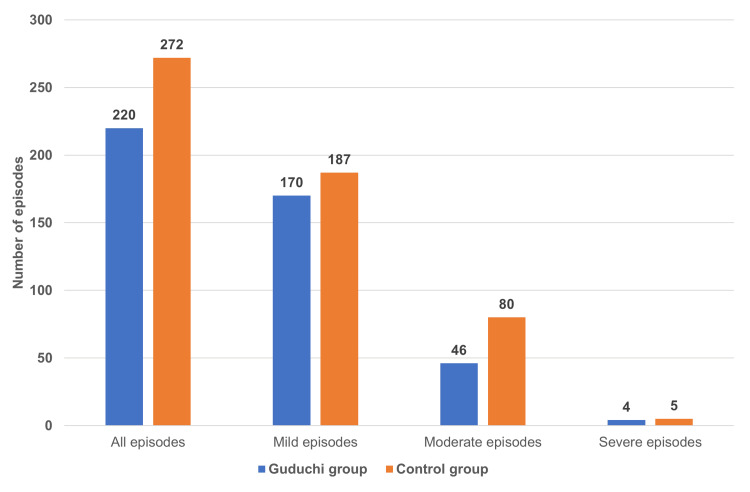
The severity of non-COVID-19-related illness Values represent the number of episodes in participants with non-COVID-19-related illnesses. A significant (p<0.05) difference was observed between the groups, with less severity in the Guduchi group compared to the control group.

A total of 492 episodes of non-COVID-19-related illness were reported in the study. Out of them, 220 (44.71%) and 272 (55.58%) episodes were reported in the Guduchi group and the control group, respectively. The number of non-COVID-19 episodes observed was significantly (p<0.05) lower in the participants of the Guduchi group compared to that of the control group. Of the 220 episodes of non-COVID-19-related illness reported in the Guduchi group, 170 (77.27%) episodes were mild, 46 (20.90%) were moderate, and four (1.81%) episodes were severe. Out of 272 episodes of the non-COVID-19 related illness reported in the control group, 187 (68.75%) episodes were mild, 80 (29.41%) episodes were moderate, and five (1.83%) episodes were severe. The severity of the non-COVID-19-related illness is presented in Figure 4.

In the Guduchi group, as per the global assessment of overall change assessed by the investigator, excellent overall efficacy was observed in 2,321 (46.33%) participants, good overall efficacy in 2,403 (47.97%) participants, satisfactory overall efficacy in 278 (5.55%) participants and poor overall efficacy was observed in seven (0.13%) participants.

## Discussion

The present study was conducted to evaluate the efficacy and safety of Guduchi Ghana Vati as a preventive remedy for healthy individuals during the COVID-19 pandemic. A total of 10,022 healthy individuals from five different districts of Rajasthan were selected. Primarily, the young population formed the major part of the study population, with an average age of 38.33±12.06 years in the Guduchi group and 35.70±11.60 years in the control group, with an almost equal distribution of male and female individuals. Incidences of occurrence of COVID-19 and other infection-related incidences/episodes were recorded through a participant-reported and investigator/researcher-verified system of data capturing in an electronic method (e-CRF). The rate of infection with COVID-19 was observed to be lower in the Guduchi group compared to the control group, but the difference was statistically non-significant. The severity of COVID-19 infection in the Guduchi group was significantly (p<0.05) lower compared to the control group, with an appreciably lesser number of participants requiring hospitalization in the Guduchi group compared to the control group. 

Similarly, there was no significant (p>0.05) difference between the groups in terms of the incidence of non-COVID-19-related illnesses, but a significant (p<0.05) difference in the severity of the non-COVID-19 related illnesses, as evident from the lesser number of episodes in the Guduchi group compared to the control group. As a preventive remedy, Guduchi Ghana Vati showed a lower rate of COVID-19 infection and other infection-related illnesses (non-COVID-19) in the study participants who took Guduchi Ghana Vati.

Guduchi, also referred to as Amruta or Giloy in Ayurveda, is classified as a Rasayana Aushadi that helps improve strength and immunity to fight against various illnesses, including infections. Formulations of Guduchi as a single herb or as polyherbal combinations were popular in Ayurveda practice not only for its potential effect in preventing infections but also as an effective therapeutic agent in the management of conditions like jwara (fever), vishama jwara, swasa, kasa, sotha, etc. Guduchi is commonly used as Guduchi Ghana Vati, a tablet prepared from a concentrated aqueous extract of Guduchi stem [13-15].

Guduchi has been reported to possess an immunity-enhancing effect in preventing and managing infections [9, 16]. The active compounds isolated from Guduchi, like giloin, giloinin, and gilosterol, along with tinosporin and berberine, have been extensively studied for their immunity-enhancing, anti-inflammatory, and anti-oxidant activities [17]. Isolated pure compounds, viz., N-methyl-2-pyrrolidone and 11-hydroxymustakone, magnoflorine, and tinocordiside, have also shown immunomodulatory effects. A recent study reported that a novel (1,4)-α-D-glucan from *Tinospora cordifolia* activates the immune system by activating macrophages via TLR6 signaling and NF-κB activation mechanisms, leading to cytokine and chemokine production [18]. Immunomodulatory protein (ImP) obtained from the dry stem powder of *Tinospora cordifolia* has been reported to augment the various immunological activities in the human body [19, 20].

Thus, Guduchi with immunomodulatory, anti-inflammatory, and anti-oxidant activity may have helped to reduce the incidence of infection-related illnesses, including COVID-19, on regular consumption for 45 days. Guduchi Ghana Vati was also found safe without producing any major adverse drug reactions.

The limitations of the study were that, since it was a community-based study conducted immediately after the announcement of the pandemic (the first wave of COVID-19) in India, followed by the first lockdown, physical contact with members of the community made it impossible to get complete, detailed health information. Also, since the study involved a large population, testing for RT-PCR was done only for selected subjects as per the prevailing policies notified by the Government of India from time to time.

## Conclusions

Results of the current study support the possible benefit of using Guduchi Ghana Vati in preventing COVID-19, as it showed a reduction in the severity of COVID-19 and further hospitalization on account of COVID-19. Additionally, the number of subjects with non-COVID-related illnesses and the number of episodes of non-COVID-19-related episodes were reduced upon usage of Guduchi Ghana Vati. Thus, the study highlights the potential benefits of Guduchi Ghana Vati for COVID-19 and non-COVID-19-related illnesses. Further randomized controlled studies involving the elderly population may lead to better clinical evidence to understand the effectiveness of this popular ayurvedic rasayana formulation.
